# Comprehensive Transcriptome and Metabolome Analyses Reveal Primary Molecular Regulation Pathways Involved in Peanut under Water and Nitrogen Co-Limitation

**DOI:** 10.3390/ijms241713308

**Published:** 2023-08-27

**Authors:** Hong Ding, Liangxiang Dai, Qing Guo, Xiaoshu Chen, Guanchu Zhang, Hao Feng, Feifei Qin, Huayuan Gao, Yang Xu, Zhimeng Zhang

**Affiliations:** 1 Shandong Peanut Research Institute, Shandong Academy of Agricultural Sciences, Qingdao 266100, China; dingpeanut@163.com (H.D.); liangxiangd@163.com (L.D.); jone007@126.com (Q.G.); guanchuzhang@126.com (G.Z.); ben0917@163.com (H.F.); jialing_300@163.com (F.Q.); 2 Peanut Research Institute, Jilin Academy of Agricultural Sciences, Gongzhuling 136100, China; 13944940275@163.com (X.C.); ghy6413@163.com (H.G.)

**Keywords:** peanut, drought stress, nitrogen deficiency, metabolome, transcriptome

## Abstract

The yield and quality of peanut (*Arachis hypogaea* L.), an oil crop planted worldwide, are often limited by drought stress (DS) and nitrogen (N) deficiency. To investigate the molecular mechanism by which peanut counteracts DS and N deficiency, we conducted comprehensive transcriptomic and metabolomic analyses of peanut leaves. Herein, 829 known differentially accumulated metabolites, 324 differentially expressed transcription factors, and 5294 differentially expressed genes (DEGs) were identified under different water and N conditions. The transcriptome analysis demonstrated that drought-related DEGs were predominantly expressed in “glycolysis/gluconeogenesis” and “glycerolipid metabolism”, while N-deficiency-related DEGs were mainly expressed in starch and sucrose metabolism, as well as in the biosynthesis of amino acid pathways. The biosynthesis, transport, and catabolism of secondary metabolites accounted for a large proportion of the 1317 DEGs present in water and N co–limitation. Metabolomic analysis showed that the metabolic accumulation of these pathways was significantly dependent on the stress conditions. Additionally, the roles of metabolites and genes in these pathways, such as the biosynthesis of amino acids and phenylpropanoid biosynthesis under different stress conditions, were discussed. The results demonstrated that different genes, metabolic pathways, and metabolites were related to DS and N deficiency. Thus, this study elucidates the metabolic pathways and functional genes that can be used for the improvement of peanut resistance to abiotic stress.

## 1. Introduction

As a significant economic and oil crop, peanut (*Arachis hydropoaea* L.) is widely planted in arid and semi-arid areas. Drought is regarded as a severe abiotic stress inhibiting peanut growth. Plants have evolved a variety of drought stress (DS) resistance and adaptation strategies, including the synthesis of osmotic protective agents and antioxidants, regulation of root growth and water absorption, and regulation of aboveground development [1,2,3,4]. To better study these strategies, transcriptomics is used to clarify molecular mechanisms in plant biology. Multiple regulatory signaling modules and functional genes related to DS have been revealed via transcriptomic analysis [5,6]. For instance, the content of active oxygen and the osmotic potential of cells were revealed as key factors related to DS resistance through PEG-6000 treatment during transcriptomic analysis [7]. Transcriptional analysis of drought-tolerant peanut varieties revealed that differentially expressed genes (DEGs) mainly participate in the photosynthesis–antenna protein, carbon metabolism, and citrate (TCA) cycle, associated with DS response [8]. Additionally, the mechanism of peanut response to DS in the middle and late growth stages has been investigated, and it was concluded that the drought tolerance of peanut involved a complex response system composed of various molecular reactions and hormones [9]. Moreover, the transcriptome analysis of peanut leaves during pod formation revealed six metabolic pathways for DS resistance, including glutathione metabolism, and starch and sucrose metabolism. This indicates that DS may trigger changes in gene expression associated with carbon metabolism, photosynthesis, and nitrogen (N) metabolism [10]. Nevertheless, a single transcriptomic technique is inadequate for clarifying complex biological processes in plants, and it is necessary to integrate information at different omics levels to identify candidate key factors.

N is an essential element for crop growth and its deficiency is commonly observed in dryland soil. Tricarboxylic acid, sugar, and amino acid accumulation was observed in barley leaves under N deficiency, and the expression of transcripts encoding for the metabolism and secondary metabolism of proteins was also upregulated for DS adaptation [11]. In rice, the transport and assimilation of N under low N conditions were through citrate and NADP accumulation and TCA cycle acceleration [12]. In peanut, transcriptomic analysis of root tissues at the seedling stage revealed that the expression of DEGs associated with amino acid and carbohydrate metabolism was enhanced under N deficiency [13]. Furthermore, DEGs related to the transportation and assimilation of nitrate, hormone signal transduction, and lignin biosynthesis were also suggested as essential regulators in peanut root growth under N deficiency [14].

Although investigations on the matter have been conducted [6,11,13], there is a limited understanding of plant responses to water and N co-limitation. Therefore, we employed transcriptomic and metabolomic analyses to clarify the mechanism of peanut adaptation to water and N co-limitation. Peanuts are extremely sensitive to water during flowering, and an inadequate water supply at this stage may lead to a serious decrease in peanut yield [15]. Therefore, we conducted greenhouse experiments for DS at the flowering stage under different N conditions and identified certain metabolic pathways, metabolites, and genes that may be important for plant adaptation to water and N dual-deficiency. Hence, this study adds to the understanding of peanut resistance mechanisms for water and N co-limitation.

## 2. Results

### 2.1. Morphological Differences between Peanuts under Different Water and N Conditions

**Figure 1:**
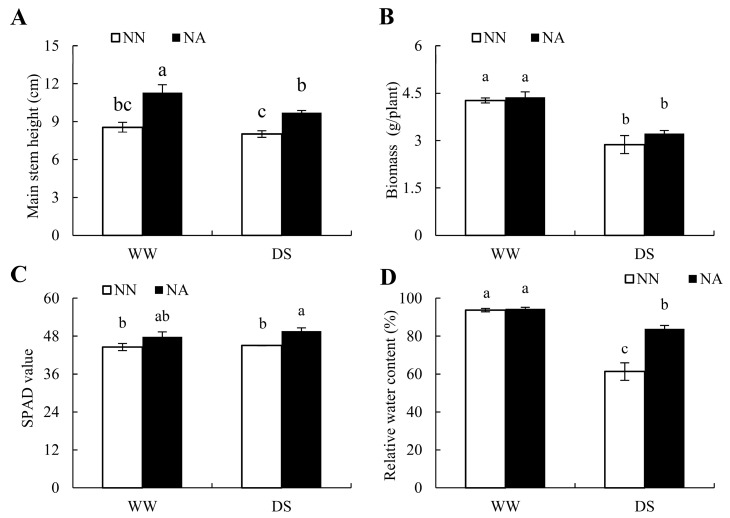
The morphological differences of peanuts under different water-N conditions. (**A**) Main stem height. (**B**) Biomass per plant. (**C**) Soil and plant analyzer development (SPAD) values. (**D**) Relative water content (RWC) of peanut leaves. The letters in the chart denote significant differences at a 0.05 probability level. WW: well-watered condition; DS: drought stress; NN: no N application; NA: N application of 90 kg ha^−1^.

Peanut morphology is highly dependent on the water and N conditions (Figure 1). Under no N application (NN), water conditions had a negligible impact on the main stem height of peanuts, while N application (NA) led to a remarkably increased main stem height of peanuts, regardless of the water conditions. Compared with NN, the increases in the main stem height of peanuts with NA under well-watered conditions (WW) and DS were 32.14% and 21.29%, respectively. On the other hand, the biomass decreased drastically under DS, while NA had negligible impacts. Compared with WW, soil and plant analyzer development (SPAD) values were increased under DS. Compared with NN, NA significantly increased the SPAD value under DS, with an increment of 9.98%. Under DS conditions, the relative water content (RWC) of peanut leaves dropped in 35.08% of DSNN and 11.17% DSNA cases, respectively, compared with those in WWNA cases. This indicates that DS inhibits the growth of peanut, and N deficiency exacerbates DS’s inhibition of peanut growth. Overall, the application of N fertilizer increased the RWC of peanut leaves under DS, and thus improved the drought resistance of peanuts.

### 2.2. Pod Yield of Peanuts under Different Water and N Conditions

**Figure 2:**
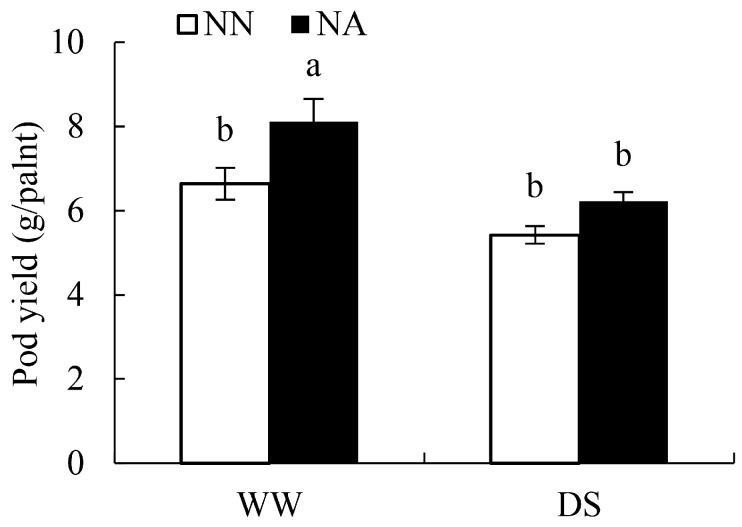
Peanut pod yield under different water and N conditions. The letters in the chart denote significant differences at a 0.05 probability level.

Compared with WWNA, DSNA reduced the pod yield of peanut by 23.20% (Figure 2), revealing the negative role of DS in peanut pod yield. Compared with WWNA, WWNN pod yield reduction was 18.18% (Figure 2), highlighting the necessary role of the N element in peanut pod yield. Under dual-deficiency, the pod yield in peanut in DSNN was 33.14% lower than in WWNA. This confirmed the hypothesis that DS inhibits the growth of peanuts and N deficiency exacerbates DS’s inhibition of peanut growth.

### 2.3. RNA Sequencing, Assembly, and Annotation of Novel Transcripts

To clary the mechanism of the effects of water and N nutrient on plant growth, we performed a transcriptomic analysis combining different water contents with or without N supply (WWNA-vs-DSNA, WWNA-vs-WWNN, WWNA-vs-DSNA, WWNN-vs-DSNN, and WWNA-vs-DSNN). In total 251,089,317 clean reads and high-quality sequences of 75.11 Gb were obtained from the 12 samples, and the minimum clean data of each sample reached 5.83 Gb. The efficiency of comparison between the reads of these samples and the reference genome ranged from 96.92% to 97.76%. The Q30 base percentages were 93.81–95.25% (Table S1). A total of 73,547 genes were identified through RNA sequencing, of which 7137 were new genes that were not well annotated. Through predictive analysis of the functional domains and protein sequences, 4872 of these genes could be annotated in one or more databases (Table 1). Overall, the sequencing results could be used for subsequent assembly and transcriptome analyses.

**Figure 3:**
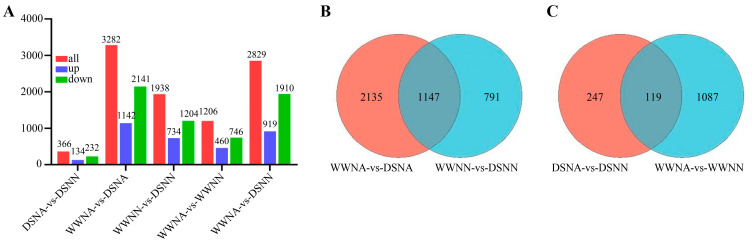
Distributions of differentially expressed genes (DEGs) from all comparisons. (**A**) The number of upregulated and downregulated DEGs in each comparison. (**B**) Venn diagram of DEGs associated with DS (WWNA-vs-DSNA and WWNN-vs-DSNN). (**C**) Venn diagram of DEGs associated with N deficiency (DSNA-vs-DSNN and WWNA-vs-WWNN). WWNA: well-watered and N application; WWNN: well-watered and no N fertilizer; DSNA: DS and N application; DSNN: DS and no N fertilizer.

Among the results, 5294 genes were differentially expressed when comparing the different combinations (Table S2). Interestingly, WWNA-vs-DSNA had the maximum number of DEGs, which was 2.7-times that of WWNA-vs-WWNN (Figure 3A), suggesting that DS might have more a serious impact on peanut leaf growth in comparison to N deficiency. A comparative analysis of DEGs showed that WWNA-vs-DSNA and WWNN-vs-DSNN had 2135 and 791 DEGs at the same N level, respectively, with the co-expression of another 1147 genes (defined as water stress response genes) from the two comparisons (Figure 3B). Under different N conditions with the same water condition, WWNA-vs-WWNN and DSNA-vs-DSNN had 1087 and 247 DEGs, respectively, with the co-expression of another 119 genes (defined as N-deficiency response genes) from the two comparisons (Figure 3C). Under water and N co-limitation (WWNA-vs-DSNN), 2829 DEGs were identified, with 919 and 1910 being upregulated and downregulated, respectively (Figure 3A). Additionally, RT-qPCR validation using randomly selected multiple genes confirmed the reliability of the transcriptome data (Figure S1). The correlation of the fragments per kilobase million (FPKM) value of RNA-seq with the RT-qPCR results determined on the basis of the Pearson correlation coefficient was significant (Figure S1), reconfirming the reliability of transcriptome sequencing data.

### 2.4. DEGs in Peanut under DS and N Deficiency

**Figure 4:**
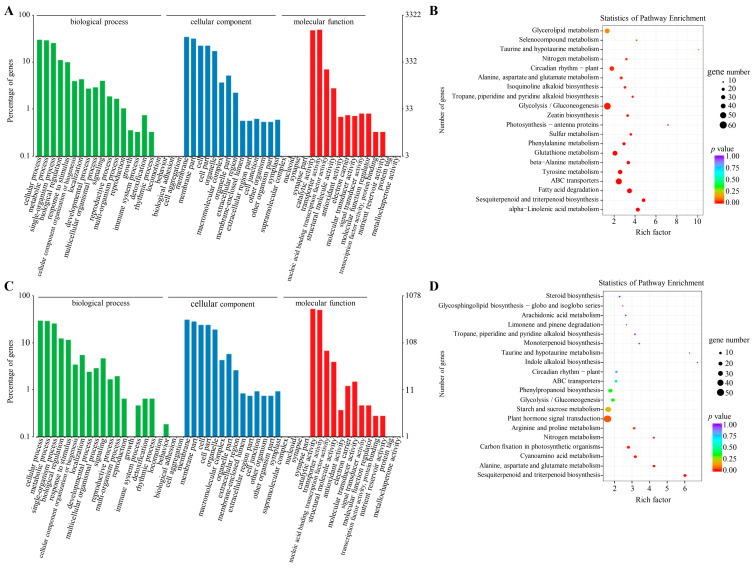
Enrichment analysis of DEGs. (**A**) GO terms for DEGs associated with DS. (**B**) KEGG pathway enrichment of DEGs related to DS. DEGs related to DS for WWNA-vs-DSNA and WWNN-vs-DSNN. Red denotes high enrichment, green represents medium enrichment, and blue denotes low enrichment. (**C**) GO terms for DEGs related to N deficiency. (**D**) KEGG pathway enrichment of DEGs related to N deficiency. DEGs related to N deficiency for WWNA-vs-WWNN and DSNA-vs-DSNN.

The functions of DEGs under DS and N deficiency were explored in the GO and KEGG databases. Under DS, 3322 genes were categorized into 47 GO terms and the 3 most abundant were “catalytic activity”, with 1639 genes; “binding”, with 1600 genes; and “membrane”, with 1150 genes (Figure 4A). The KEGG enrichment of the genes related to DS response suggested that they were dominantly enriched within glycolysis/gluconeogenesis pathways, ABC transporters, fatty acid degradation, glutathione metabolism, and glycerolipid metabolism (Figure 4B), suggesting that energy and lipid metabolism are key metabolic pathways associating peanut leaf response to DS.

Under N deficiency, 1078 DEGs were divided into 46 GO terms depending on cellular components, molecular functions, and biological processes (Figure 4C). For the biological process, 318, 313, and 276 genes were related to the “cellular process”, “metabolic process”, and “single-organism process”, respectively. For the cellular component, 332 and 304 genes were related to the “membrane” and “membrane part”, respectively. In the category of molecular function, genes related to “binding” (563 genes) and “catalytic activity” (537 genes) were dominant. In addition, 501 DEGs were assigned to 113 KEGG pathways, and 20 pathways were determined on the basis of the *p*-value (*p* < 0.05) (Figure 4D). Herein, 54, 39, 30, 28, and 27 genes were correlated with plant hormone signal transduction, starch and sucrose metabolism, carbon metabolism, biosynthesis of amino acids, and phenylpropanoid biosynthesis, respectively.

**Figure 5:**
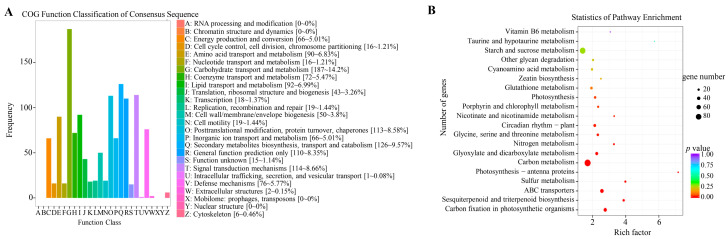
Enrichment analysis of DEGs related to water and N co-deficiency (WWNA-vs-DSNN). (**A**) COG function classification of DEGs. (**B**) KEGG pathway enrichment of DEGs. Red denotes high enrichment, green represents medium enrichment, and blue denotes low enrichment.

Under water and N co-limitation, COG function annotation showed that transport and metabolism of carbohydrates and biosynthesis, transport and catabolism of secondary metabolites accounted for 14.2% and 9.6% of 1317 DEGs, respectively (Figure 5A). The number of DEGs rich in carbon metabolism regulatory pathways were determined via KEGG enrichment analysis (Figure 5B), including carbon metabolism (80), starch and sucrose metabolism (73), carbon fixation in photosynthetic organisms (39) and glyoxylate and dicarboxylate metabolism (34), while the number of DEGs in N-related metabolic pathways such as glutathione (28) and N metabolism (17) was lower. The number of DEGs in the biosynthesis of secondary metabolites pathways such as phenylpropanoid biosynthesis (46) and flavonoid biosynthesis (16) was also determined. Overall, carbon metabolism and secondary metabolites pathways may play important roles for peanuts for water/N dual-deficiency adaptation.

### 2.5. Analysis of Genes Encoding Transcription Factors

**Figure 6:**
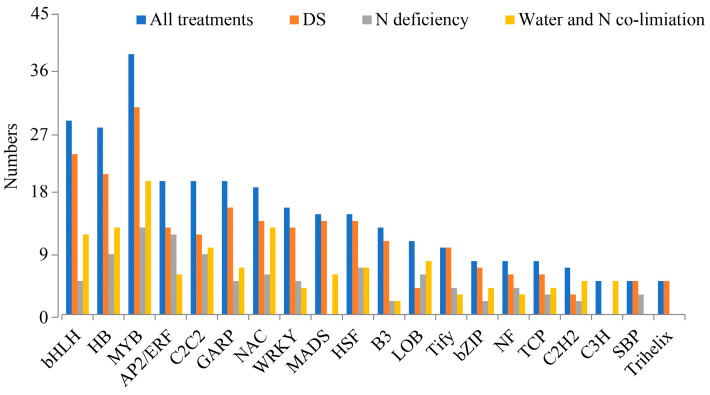
The transcription factors of DEGs for different treatments. Transcription factors related to DS for WWNA-vs-DSNA and WWNN-vs-DSNN; N deficiency for WWNA-vs-WWNN and DSNA-vs-DSNN; water and N co-limitation for WWNA-vs-DSNN.

Transcription factors play essential regulatory roles in the expression of stress-responsive genes. Herein, 324 differentially expressed transcription factors were identified, mainly belonging to MYB (v-myb avian myeloblastosis viral oncogene homolog, 39), bHLH (basic helix-loop-helix transcription factor, 29), HB (homeobox, 28), AP2/ERF (apetala 2/ethylene-responsive element binding factor, 20), C2C2 (20), and GARP (GA binding protein transcription factor, 20), comprising over 30 gene families (Figure 6). As observed, MYB was the highest-expressed transcription factor family among all groups. Regarding transcription factors, 109, 245, and 146 were identified under N deficiency, DS, and water and N co-limitation, respectively. Moreover, the MYB, bHLH, HB, GARP, and heat shock factor (HSF) transcription factor families were identified under DS. Under N deficiency, the highly expressed transcription factor families included MYB, AP2/ERF, C2C2, HB-HD-ZIP, and HSF. Under water and N co-limitation conditions, MYB, HB, NAC, bHLH, C2C2, and NAC were highly expressed.

### 2.6. Metabolic Analysis of Peanut Responses to Different Stress Conditions

**Figure 7:**
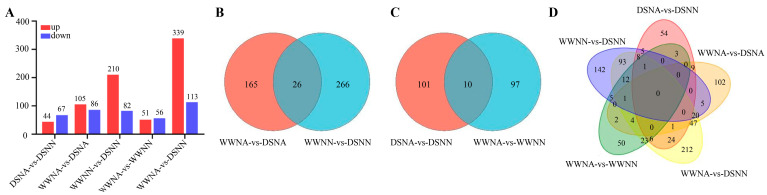
Distributions of differentially accumulated metabolites (DAMs) for all comparisons. (**A**) The number of upregulated and downregulated DAMs. (**B**) Venn diagram of DAMs related to DS (WWNA-vs-DSNA and WWNN-vs-DSNN). (**C**) N deficiency (WWNA-vs-WWNN and DSNA-vs-DSNN) and (**D**) all treatments.

Metabolites are directly involved in the physiological and biochemical processes of various plants, and changes in the metabolome under abiotic stress may reflect the plant response. Therefore, we performed metabolic analyses combining different water contents, with or without N supply. A total of 3867 metabolites were identified, of which 829 were differentially expressed when comparing the different combinations (Table S2). Under DS, 191 (105 upregulated, 86 downregulated) and 292 (210 upregulated, 82 downregulated) metabolites were differentially accumulated in WWNA-vs-DSNA and WWNN-vs-DSNN, respectively. Under N deficiency, 107 (51 upregulated, 56 downregulated) and 111 (44 upregulated, 67 downregulated) metabolites were differentially accumulated in WWNA-vs-WWNN and DSNA-vs-DSNN, respectively. Additionally, 339 and 113 metabolites were upregulated and downregulated, respectively, in WWNA-vs-DSNN (Figure 7A).

Additionally, 457 metabolites were accumulated in WWNA-vs-DSNA and WWNN-vs-DSNN (Figure 7B), possibly attributable to the DS response of peanut leaves; therefore, these metabolites were defined as DS-related metabolites. On the other hand, the 208 metabolites that accumulated in both WWNA-vs-WWNN and DSNA-vs-DSNN were defined as N-deficiency-related metabolites (Figure 7C). Furthermore, 50, 54, 102, 142, and 212 metabolites were accumulated in WWNA-vs-WWNN, DSNA-vs-DSNN, WWNA-vs-DSNA, WWNN-vs-DSNN, and WWNA-vs-DSNN, respectively (Figure 7D).

### 2.7. Analysis of Metabolic Pathways Related to Differentially Accumulated Metabolites (DAMs)

**Figure 8:**
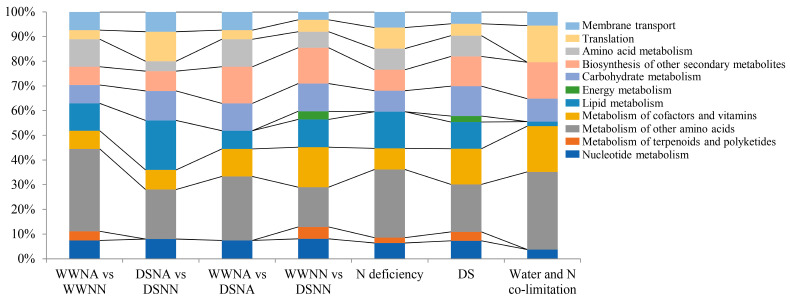
KEGG classifications of DAMs.

Metabolomics was employed to clarify the response mechanism of plant stresses, as tiny changes in gene and protein expressions could be magnified at metabolic levels. We found that more known differential metabolites, especially those related to carbohydrate metabolism, were accumulated in DSNA-vs-DSNN compared with WWNA-vs-WWNN (Figure 8), indicating that the combination with DS led to the accumulation of more metabolites in peanut leaves under N-deficiency conditions. Likewise, more well-established differential metabolites, especially those related to the biosynthesis of other secondary metabolites, were accumulated in WWNN-vs-DSNN compared with WWNA-vs-DSNA. These results suggested that the combination with N deficiency led to the accumulation of more metabolites in peanut leaves under DS conditions. Additionally, KEGG analysis revealed that abundant metabolites were assigned to pathways related to the biosynthesis of other secondary metabolites and energy metabolism under DS (WWNA-vs-DSNA and WWNN-vs-DSNN), while a large portion of metabolites were assigned to the pathways related to the metabolism of amino acids and carbohydrates under N deficiency (WWNA-vs-WWNN and DSNA-vs-DSNN). It is noteworthy that under water and N co-limitation, significant enrichment of differential metabolites was observed in the flavone and flavonol biosynthesis, as well as phenylpropanoid biosynthesis, in peanut leaves, suggesting that flavonoids and corresponding metabolites may play a key role in peanut stress adaptation under water/N dual-deficiency.

### 2.8. Integrated Analysis of DEGs and DAMs

**Figure 9:**
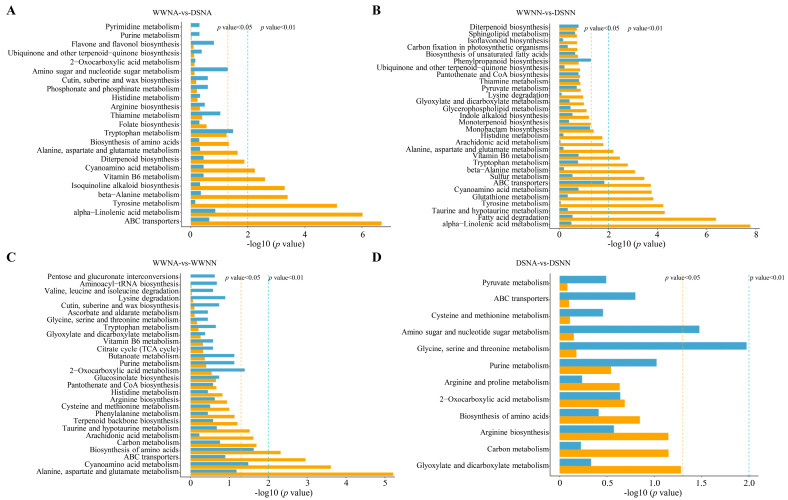
KEGG pathway analysis. Histograms of joint KEGG enrichment *p*-value between DAMs and DEGs in WWNA-vs-DSNA (**A**), WWNN-vs-DSNN (**B**), WWNA-vs-WWNN (**C**), and DSNA-vs-DSNN (**D**). “Meta” and “Gene” represent the KEGG pathways enriched by DAMs and DEGs, respectively. The yellow line denotes significance (*p* < 0.05).

An interaction network of the five groups was developed to investigate the correlation of DAMs and DEGs. These DEGs were subjected to a Pearson correlation coefficient (PCC) analysis, with a PCC >0.8. The key signal transduction and biochemical pathways involved in DAMs and DEGs were identified by mapping DEGs and DAMs to the KEGG pathway database. KEGG analysis between DEGs and DAMs under DS revealed 23 co-enrichment pathways in WWNA-vs-DSNA (Figure 9A), among which amino sugar and nucleotide sugar metabolism, and amino acid metabolism were dominant between DAMs and DEGs. Additionally, 52 KEGG co-enrichment pathways were identified in WWNN-vs-DSNN, and the top 30 KEGG pathways enriched according to the *p*-value of the genes are shown in Figure 9B: the phenylpropanoid biosynthesis and amino acid metabolism were significantly enriched between DAMs and DEGs.

In total, 28 KEGG co-enrichment pathways were identified in WWNA-vs-WWNN, and the carbon metabolism and biosynthesis amino acids were significantly enriched between DAMs and DEGs (Figure 9C). Twelve co-enrichment pathways were identified in DSNA-vs-DSNN (Figure 9D), while pathways related to the metabolisms of amino sugar and nucleotide sugar, and the metabolisms of glycine, serine, and threonine were remarkably enriched. Additionally, the carbohydrate metabolism and amino acids biosynthesis were the main enriched pathways under N deficiency.

Well-established carbohydrate and amino acid metabolic processes form the basis of plant growth. Under DS and N deficiency, 13 metabolites changed due to significantly differential expression of 88 genes in the biosynthesis of amino acids (Table 2 and Table S3). N-Acetyl-D-glucosamine, N-Acetylmuramate, and D-Fructose changed due to significantly differential expressions of 42 genes in amino sugar and nucleotide sugar metabolism (Table 2 and Table S3).

**Figure 10:**
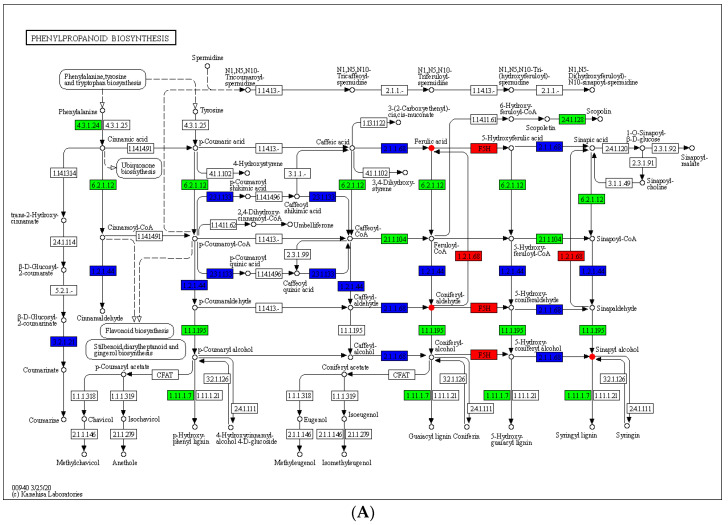

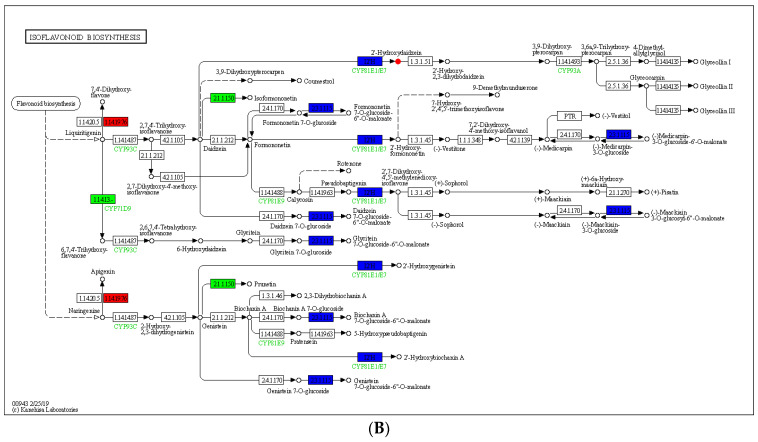
Analysis of the DEGs and differentially accumulated flavonoids under water and N co−limitation. KEGG enriched pathways for (**A**) the phenylpropanoid biosynthesis pathway and (**B**) the isoflavonoid biosynthesis pathway. The boxes in the figure represent gene products, and the circles represent metabolites. The gene products/metabolites with red/green background boxes belong to the differential genes/metabolites detected in this study, where red represents upregulated genes/metabolites and green represents downregulated genes/metabolites. All gene products with blue background boxes belong to genes/metabolites that are both upregulated and downregulated.

Under water and N co-limitation, two pathways of the biosynthesis of other secondary metabolites (phenylpropanoid biosynthesis and isoflavonoid biosynthesis) were found to be highly enriched, suggesting that N deficiency stimulated the accumulation of secondary metabolites under DS. A total of 3 metabolites and 46 key genes were simultaneously mapped to the phenylpropanoid biosynthesis pathway (ko00940), and 11 genes and 1 metabolite were mapped to the isoflavonoid biosynthesis pathway (ko00943). These results further demonstrated the key role of flavonoids in peanut resistance to water/N dual-deficiency (Figure 10, Table S3).

## 3. Discussion

### 3.1. Peanut Growth under Different Water and N Conditions

DS and N deficiency are essentially the key limiting factors for peanut growth [13,16], and the combination of nutrient deficiency and DS exacerbates adverse effects [17]. It has been demonstrated that appropriate N application can alleviate the adverse effects of DS on plant growth [18,19]. In this study, both DS and N deficiency alone significantly reduced the biomass and RWC of peanut leaves and inhibited peanut growth (Figure 1). On the other hand, the adverse effects of dual-deficiency on peanut growth and pod yield were more severe (Figure 1 and Figure 2). Both watering and N fertilizer applications rescued plant growth and pod yield under water and N co-limitation conditions. Additionally, the SPAD value of leaves was affected by N deficiency but not DS; this was related to the fact that N application provided necessary proteins for chlorophyll synthesis. Under DS conditions, N fertilizer application increased the biomass, RWC, and pod yield by 12.10%, 36.84%, and 14.87%, respectively. Overall, N fertilizer application mitigated the adverse effects caused by DS, which is consistent with a previous study [17].

### 3.2. Major Pathways Related to Response to Water and N Deficiency

Water and N nutrition regulate all aspects of plant growth, especially leaf development. Here, we employed transcriptome and metabolome analysis to investigate the regulation mechanism of peanut leaves under water and N dual-deficiency. Inter-group comparisons revealed the difference of molecular distribution under different stresses. Previous transcriptome studies showed that the DEGs in peanut related to biosynthesis of secondary metabolites, photosynthesis antenna proteins, and N metabolism were associated with DS response [7,8,9,10]. The genes related to ABA-signaling were considered a determinative factor for drought tolerance in peanuts [7,9]. Some studies also investigated the N-deficiency-related pathways in peanut roots [11,12], which showed that DAMs were mainly involved in carbon and N metabolism [11]. In this study, 5294 DEGs and 829 DAMs were identified via using multi-omics analysis. Some DEGs under DS were involved in photosynthesis antenna proteins and N metabolism (Figure 4B), which was consistent with the above-described research. Nonetheless, we found some energy and lipid metabolism-related DEGs in peanut leaves under DS, while the ABA-signaling-related genes were not enriched. In previous studies, peanuts were treated with PEG-6000 to simulate DS [7,8], which could be one possible reason for these inconsistent results. Furthermore, the peanut variety, the stress treatment time, and sampled tissues may be also be related to the different gene expressions. Consistent with other studies on N-deficiency [11,12], we also found that N-deficiency-related DEGs and DAMs were mainly enriched in starch and sucrose metabolism, biosynthesis of amino acids, and hormone signal transduction (Figure 4D, Figure 8).

Metabolites directly participate in biochemical and physiological processes in plants, leading to a series of reactions under environmental stresses [12,20]. As the core part of the physiological metabolism in plants, primary metabolites play a dominant role in the growth and abiotic stress resistance of plant. Here, the analysis of DEGs and DAMs led us to focus on the metabolic pathways of carbohydrates and amino acids (Figure 9). Indeed, primary metabolites, such as amino acids and sugars have been demonstrated to be key players in stress resistance [21]. Additionally, amino acid metabolism, especially serine metabolism, is closely related to abiotic stress response and N metabolism [22]. As an auxin precursor in higher plants, L-tryptophan could relieve the adverse effects of drought on maize and wheat [23,24]. In this study, the level of L-serine decreased significantly under N deficiency and DS (Table 2), resulting in significant downregulation of two genes (*Arahy.HSR92P* and *Arahy.BCZ98G*) related to serine hydroxymethyl transferase in WWNN-vs-DSNN (Table S3). Although L-tryptophan metabolites increased significantly under DS, the enrichment under water and N co-limitation conditions was lower than that under DS alone.

The carbohydrate pathway provides energy for plant growth and contributes to abiotic stress tolerance [25,26]. For instance, the levels of fructose were significantly enhanced in maize under DS conditions [27,28]. D-fructose in peanut leaves was enriched in DS, as well as N deficiency. Indeed, water and N deficiencies inhibit the TCA cycle, resulting in hindered glucose oxidation [12,13]. Therefore, it was deduced that DS and N deficiency inhibit the TCA cycle in peanut leaves and subsequently repress the accumulation of carbohydrate metabolites. Hexokinase (HXK) is a sugar-phosphorylating enzyme involved in sugar metabolism, and *MdHK1/2/3/5* were downregulated in apple leaves after six days of DS treatment [29]. *Arahy.*86IGSK and *Arahy.*YT3TY8 were related to HXKs. We found that *Arahy.86IGSK* was downregulated in response to DS and *Arahy.YT3TY8* was downregulated in response to water and N co-limitation (Table S3). Based on these results, we suspect that the downregulation of HXK-related genes may be the reason for fructose aggregation under DS.

Secondary metabolites also play a key role in plant resistance to abiotic stresses. Previous studies have established the essential role of secondary metabolites in plant abiotic stress responses [30,31]. The contents of phenol and flavonoid in tomato under DS increased drastically after fungal treatment, resulting in enhanced drought resistance [32]. Alongside that, the tolerance of wild soybean to N deficiency is related to its ability to enhance the synthesis of favorable secondary metabolites under low N conditions [33]. The relationships between secondary metabolites in peanut leaves under water and N co-limitation can help in understanding the correlation of physiology and abiotic stress in peanuts. In this study, the DAMs of peanut were classified into various comparisons, and most differential metabolites related to water and N co-limitation were assigned to the biosynthesis of other secondary metabolites. Four secondary metabolites related to flavonoid synthesis, including coniferyl aldehyde and 2′-hydroxyclaidzein, were significantly enriched under water and N co-limitation (Figure 10). Five secondary metabolites were significantly different in WWNN-vs-DSNN, while the other comparisons had negligible differences in secondary metabolites enrichments (Table S4). Overall, flavonoid and isoflavone metabolism plays an important role in DS response, and N deficiency exacerbates the changes of secondary metabolites in peanut leaves.

### 3.3. Transcription Factors Related to Response to Water and N Deficiency

Transcription factors are important regulatory factors of the response to abiotic stresses [34]. In this study, various transcription factor families were identified, and many of them were associated with resistance to DS or N deficiency (Figure 6). The most enriched transcription factors were MYB, bHLH, and HB. MYB regulates stomatal movement through ABA signaling, owing to drought/oxidative stress regulation in rice and *Arabidopsis* [35,36]. Thirty-one, thirteen, and twenty MYB genes were identified under DS, N deficiency, and water and N co-limitation, respectively, indicating that MYB transcription factors play essential roles in DS and N-deficiency response. The bHLH transcription factors also play a regulatory role in DS response, and overexpression of several bHLH members improved the drought resistance of plants [37,38,39]. FtbHLH3 promotes drought tolerance via an ABA-dependent pathway in *Arabidopsis* [40]. In addition to bHLH transcription factors, AP2/ERF also plays a regulatory role in N-deficiency response. Previous studies have shown that AP2/ERF plays a key role in response to drought and salt stresses [41,42], while N-deficiency response has not been fully understood. In summary, these transcription factors might be related to DS and N-deficiency response and play an important role in regulating the resistance of peanut to the compounded stresses of DS and N deficiency.

## 4. Materials and Methods

### 4.1. Experimental Design

“Yuhua 9326”, a drought-resistant peanut variety, was employed in this study, and a pot experiment under 16 h/8 h light–darkness cycle was executed (28 °C/22 °C). The test pots had a diameter of 23 cm and a height of 25 cm, with 5.5 kg of soil and 3 peanuts in each pot. The soil used was a light loam soil, with a medium N supply level (alkali-hydrolyzable N = 56.7 mg kg^−1^). The water conditions in the flowering stage were set to be normal water supply (well-watered, WW; 75 ± 5% field water capacity) or DS (45 ± 5% field water capacity). The N fertilizer application was set to no N (NN) or N application (NA; 90 kg ha^−1^). Except for N fertilizer, calcium superphosphate (450 kg ha^−1^) and potassium sulfate (300 kg ha^−1^) were applied in each treatment. In the case of DS, the water content was reduced to 45% of the field water capacity and the samples were collected five days after the stress [43]. After DS treatment, a well-watered supply was restored for all groups until the harvest period.

Four groups were designed in this study: (1) control group (well-watered with N application, WWNA); (2) the drought group (DS with N application, DSNA); (3) the N-deficiency group (well-watered without N application, WWNN); (4) and water and N co-limitation group (DS without N application, DSNN). Each group had at least three biological replicates and each replicate had three leaf samples isolated from different plants.

### 4.2. Determination of Morphological Traits and Pod Yield of Peanuts

The main stem height was defined as the distance from the cotyledon node to the growth point. First, fresh plants were dried in an oven at 75 °C for no less than 48 h. The soil and plant analyzer development (SPAD) value of the third fully expanded leaves of the main stem were measured using a portable chlorophyll meter (SPAD 502). Additionally, the relative water content (RWC) of the third fully expanded leaves in the main stem was determined: RWC% = (fresh leaf mass − dry leaf mass)/(saturated fresh leaf mass − dry leaf mass) × 100%. The pod yield was obtained from peanuts for each pot at the final harvest. The pods were air-dried to about 8% humidity, and the mass of the pods was measured using an electronic balance.

### 4.3. RNA Extraction, Library Construction, and RNA Sequencing

Total RNA extraction from leaves was achieved utilizing Trizol reagent (Invitrogen) [44], and qualified RNA samples were selected for high throughput sequencing. First, mRNA was isolated using magnetic beads with Oligo (dT) and then randomly broken. With segmented mRNA as the template, double-stranded cDNA was synthesized in the presence of reverse transcriptase, followed by library enrichment by PCR amplification. After quality inspection, PE150 sequencing was executed utilizing the Illumina NovaSeq 6000 sequencing platform.

### 4.4. Transcriptomic Analysis

The raw paired-end reads were trimmed and quality controlled using SeqPrep (https://github.com/jstjohn/SeqPrep accessed on 24 August 2023) and Sickle (https://github.com/najoshi/sickle/ accessed on 9 September 2022) with default parameters. Then, clean reads were separately aligned to the peanut reference genome Tiffrunner (https://data.legumeinfo.org/Arachis/hypogaea/annotations/Tifrunner.gnm2.ann1.4K0L/ on accessed 2 October 2022) from the Peanut Genome Database (https:/www.peanutbase.org/ accessed on 2 October 2022). The mapped reads of each sample were assembled using StringTie (https://ccb.jhu.edu/software/stringtie/index.shtmlt=example/ accessed on 14 November 2022) [45]. To identify differential expression genes (DEGs) for different combinations, the expression level of each transcript was calculated according to the transcripts-per-million-reads method. RSEM (http://deweylab.biostat.wisc.edu/rsem/ on 17 November 2022) [46] was used to quantify gene abundances. DEGs with |log_2_FC| > 1 and *p*-value < 0.05 (DESeq2) were considered significantly differentially expressed genes. In addition, functional enrichment analyses, including GO and KEGG, were performed to identify which DEGs were significantly enriched in GO terms, and metabolic pathways at the Bonferroni-corrected *p*-value < 0.05 were compared with the whole-transcriptome background. GO functional enrichment and KEGG pathway analyses were carried out using Goatools (https://github.com/tanghaibao/Goatools/ on accessed 21 November 2022) and KOBAS (http://kobas.cbi.pku.edu.cn/home.do/ on accessed 25 November 2022) [47].

### 4.5. Metabolite Extraction and Analysis

A freeze-dried sample (50 mg) was weighed and added to 1000 μL of extraction solution (methanol: acetonitrile: water = 2:2:1 in a volume ratio, with 20 mg/L internal standard), and the solution was homogenized and mixed. Then, the mixture was homogenized in a ball mill for 10 min with a 45 Hz grinder, and sonicated in an iced water bath for 10 min, followed by incubation at −20 °C for 60 min. It was then centrifugation at 12,000 rpm at 4 °C for 15 min. About 500 μL of the supernatant was obtained and dried in an EP tube in a vacuum concentrator, followed by the addition of 160 μL of extraction solution (the volume ratio of acetonitrile and water was 1:1) to dissolve the dried metabolites. The samples were then swirled for 30 s, followed by sonication in an iced water bath for 10 min and centrifugation at 12,000 rpm at 4 °C for 15 min. After that, 120 μL of the supernatant was transferred to a LC/MS glass vial. Finally, 10 μL of each sample was collected as a quality-control sample for analysis via ultra-high performance liquid chromatography tandem quadrupole time of flight mass spectrometry (UHPLC–QTOF–MS).

Metabolite separation was achieved via chromatography using Acquity UPLCI-Class PLUS (Waters, Milford, MA, USA) and a mass spectrometer (Xevo G2-XS QTOF, Waters, Milford, USA), with an Acquity UPLC HSS T3 chromatographic column (1.8 μm 2.1 × 100 mm, Waters, USA). Solvents A and B were aqueous and acetonitrile (1:1, *v*/*v*) solutions of 0.1% formic acid, respectively. The elution was as follows: 0 min, 2% B; linearly increasing to 98% within 10 min, and maintained at 98% for 3 min; Phase B ratio decreasing to 2% after 13–13.1 min, and balancing at 5% for 15 min. The column temperature and the flow rate were kept at 40 °C and 0.35 mL/min, respectively. The injection volume for the positive and negative ion was 1 μL. The negative and positive modes of ion injection voltage fluctuations were −2000 V and 2500 V, respectively. The low and high collision energy were 2 V and 10–40 V, respectively. The mass range was 50–1200 *m*/*z*.

The raw data obtained were processed using Progenesis QI software, in terms of peak extraction and alignment. The repeatability of the samples was determined via Spearman correlation and principal component analyses. The classification and pathway information of the compounds identified in this study were searched in HMDB, KEGG, and lipidmaps databases. *p* values were determined through T-tests. An orthogonal projections to latent structures-discriminant analysis (OPLS-DA) was executed utilizing the R language package. The difference multiple, the *p*-value, and VIP value (variable importance in the projection) of the OPLS-DA model were employed for the screening of differential metabolites based on the following criteria: FC > 1, *p* < 0.01, and VIP > 1 [48].

### 4.6. Verification of DEGs via RT-qPCR

Seven genes were randomly selected, and the transcriptome sequencing results were validated using RT-qPCR. Primer 5.0 software was employed for the design of amplification primers (Table S5). Each sample was subjected to three biological replications. *Actin* gene homogenization was involved, and the 2^–ΔΔCt^ method was used to determine the relative expression of DEGs [49].

### 4.7. Data Analysis

A variance analysis and difference significance analysis were conducted using SPSS and Duncan’s method [50], respectively. Additionally, multiple comparisons at a probability level of 5% were performed using Duncan’s multiple range test.

## 5. Conclusions

Under DS and N-deficiency conditions, the metabolic processes in peanut leaves were significantly changed. The pathways related to the biosynthesis of other secondary metabolites and energy metabolism were mainly affected by DS, while those related to carbohydrate and amino acid metabolism were mainly affected by N deficiency. Some pathways related to secondary metabolites, such as isoflavonoid and phenylpropanoid were enriched under water and N co-limitation, which may play important roles in dual-deficiency stress adaptation. The transcriptomic analysis revealed that the key genes related to such metabolic pathways were also remarkably changed. Through the combined analyses of transcriptome and metabolome, we revealed some important metabolic pathways, DAMs, and DEGs related to DS and N-deficiency responses. In summary, this study clarifies the mechanism of peanut’s resistance to DS, N deficiency, and water and N co-limitation, and provides a theoretical basis for peanut breeding in DS conditions.

## Figures and Tables

**Table 1:** Functional annotation of the peanut transcriptomes in the six public databases involved.

Annotated Databases	New Gene Number
Clusters of Orthologous Groups (COG)	363
Gene Ontology (GO)	2300
Kyoto Encyclopedia of Genes and Genomes (KEGG)	1754
Clusters of orthologous groups for eukaryotic complete genomes (KOG)	1302
Protein family (Pfam)	1838
Swiss-Prot	1431
NCBI non-redundant protein sequences (nr)	4850
All	4872

**Table 2:** The abundance of DAMs related to the biosynthesis of amino acid, and amino sugar and nucleotide sugar metabolism, pathways.

Pathway	Metabolite	WWNA	WWNN	DSNA	DSNN
Biosynthesis of amino acid	N-Acetyl-L-glutamate 5-semialdehyde	504.23	1632.39	492.14	988.54
	3-(Imidazol-4-yl)-2-oxopropyl phosphate	5094.62	4686.56	3757.68	3952.69
	N-(L-Arginino)succinate	461.31	670.57	1281.64	1285.71
	(S)-2-Aceto-2-hydroxybutanoate	214.09	259.90	291.11	368.21
	3-Dehydroquinate	222.21	343.09	436.01	454.96
	L-Valine	129.76	88.94	144.84	100.84
	N-Succinyl-2-L-amino-6-oxoheptanedioate	950.74	1838.30	1046.38	1765.93
	Oxoglutaric acid	2040.25	4760.55	1137.81	3142.27
	L-Aspartic Acid	678.33	751.24	1066.28	1040.05
	L-Serine	148.90	55.72	54.25	20.25
	L-Tryptophan	227.47	78.22	583.69	463.70
	alpha-Isopropylmalate	283.29	51.00	277.22	149.81
Amino sugar and nucleotide sugar metabolism	N-Acetyl-D-glucosamine	406.34	324.57	651.31	314.02
	N-Acetylmuramate	23.25	19.04	74.21	10.15
	D-Fructose	495.44	541.21	604.81	641.01

Notes: WWNA: well-watered and N application; WWNN: well-watered and no N fertilizer; DSNA: DS and N application; DSNN: DS and no N fertilizer.

## Data Availability

The transcriptome data have been deposited in the National Center for Biotechnology Information (NCBI) BioProject database with valid accession number PRJNA980668, and the metabolome data have been deposited in the CNGB Sequence Archive (CNSA) of China National GeneBank DataBase (CNGBdb) with accession number CNP0004572 (https://db.cngb.org/search/metabolize/METM0000127/, accessed on 24 August 2023).

## Supplementary Materials

The following supporting information can be downloaded at: https://www.mdpi.com/article/10.3390/ijms241713308/s1.
