# Supplementary material for: Comprehensive Transcriptome and Metabolome Analyses Reveal Primary Molecular Regulation Pathways Involved in Peanut under Water and Nitrogen Co-Limitation
Source: Int J Mol Sci. 2023 Aug 27;24(17):13308. doi: 10.3390/ijms241713308 (PMC10487698; doi:10.3390/ijms241713308)

Figure S1. RT-qPCR validation of the DEGs in peanut leaves. Shown are their relative expression levels; bars are the standard deviation. The *AhACT11* gene was used as an internal control to normalize the expression data. The Pearson correlation coefficient was used to calculate the correlation coefficient between the TPM value of RNA-seq and qRT-PCR value for each gene and fold change (WWNN/WWNA, DSNA/WWNA and DSNN/WWNA) value of TPM and normalized qPCR for 7 genes. The results indicate a strong correlation between these two approaches. The correlation coefficient (R^2) is indicated in this figure.

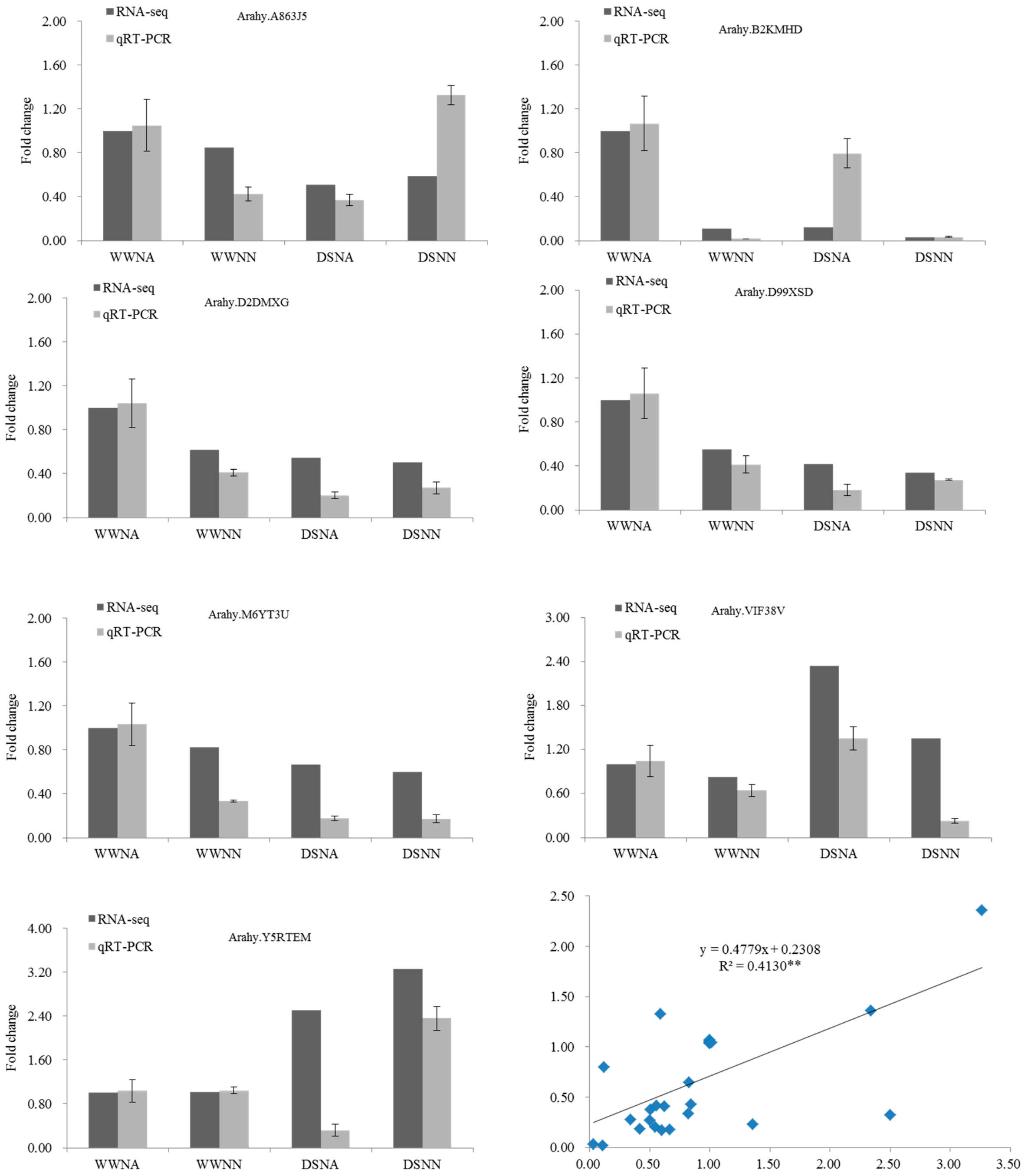

Supplement: Supplementary file 1 [file ijms-24-13308-s001.zip › Figure S1.pdf]
