# Supplementary material for: Comprehensive Transcriptome and Metabolome Analyses Reveal Primary Molecular Regulation Pathways Involved in Peanut under Water and Nitrogen Co-Limitation
Source: Int J Mol Sci. 2023 Aug 27;24(17):13308. doi: 10.3390/ijms241713308 (PMC10487698; doi:10.3390/ijms241713308)
Supplement: Supplementary file 1 [file ijms-24-13308-s001.zip › Table S1.pdf]

Table S1. Sequencing data statistics under different conditions.

Samples	Clean reads	Clean bases	GC Content	%≥Q30
DSNA1	20,450,923	6,116,316,278	45.06%	94.18%
DSNA2	20,323,995	6,078,686,832	45.31%	94.17%
DSNA3	21,719,624	6,494,300,110	45.15%	94.26%
DSNN1	21,168,374	6,332,165,702	44.97%	94.07%
DSNN2	22,109,833	6,614,792,124	44.91%	94.38%
DSNN3	20,477,772	6,125,333,102	44.97%	94.20%
WWNA1	22,246,029	6,656,529,770	45.25%	94.26%
WWNA2	19,504,699	5,832,861,280	45.06%	94.36%
WWNA3	20,445,315	6,114,429,120	45.40%	93.81%
WWNN1	20,579,949	6,156,799,436	44.92%	94.31%
WWNN2	20,319,087	6,082,469,764	45.13%	95.25%
WWNN3	21,743,717	6,508,347,362	45.06%	94.12%
