# Supplementary material for: Comprehensive Transcriptome and Metabolome Analyses Reveal Primary Molecular Regulation Pathways Involved in Peanut under Water and Nitrogen Co-Limitation
Source: Int J Mol Sci. 2023 Aug 27;24(17):13308. doi: 10.3390/ijms241713308 (PMC10487698; doi:10.3390/ijms241713308)
Supplement: Supplementary file 1 [file ijms-24-13308-s001.zip › Table S5.pdf]

Table S5. RT-qPCR primers used in this study.

Gene ID	Description	Primer sequence (5′–3′)
Actin		F: TTGGAATGGGTCAGAAGGATGC
		R: GCTTCTTACTGAGGCACCACT
Arahy. A863J5	Glutamine synthetase leaf isozyme	F: GAATCGATGTGCGCAGTAAATCA
		R: TTGTTTGTAGGGATAGGCTCACC
Arahy. B2KMHD	Inducible nitrate reductase [NADH] 2	F: AATCACGCCCATTTACCAAGTTG
		R: TGTATTCCCACCCTTCTCTTTGG
Arahy. D2DMXG	Glyceraldehyde-3-phosphate dehydrogenase A	F: TCCGCAACTCATCATCTAGCTAC
		R: CTTTCCTGTATCCTCCACTGCTT
Arahy. D99XSD	Probable ribose-5-phosphate isomerase 3	F: TCATCATCCTTAACCCTGCCATC
		R: GTCTGTGTGTGTTGTTGTTGGG
Arahy. M6YT3U	Sedoheptulose-1,7-bisphosphatase	F: CTTTCAAGCACTCTCCTCCTTCT
		R: GCCCTTTGCCTTTGAAACCTTAT
Arahy. VIF38V	Fructose-1,6-bisphosphatase	F: ATACTGTGTTGTTTTTGACCCGC
		R: AATAACCAGCTGCCAACATGTTC
Arahy. Y5RTEM	Beta carbonic anhydrase 5	F: GAAGAGATGGCGTGATGAATTGG
		R: AGCCATATTCCGCACATCAGTAT
